# Development and validation of a nomogram to predict long-term cancer-specific survival for patients with osteosarcoma

**DOI:** 10.1038/s41598-023-37391-8

**Published:** 2023-06-23

**Authors:** Yali Yu, Shaohua Wang, Jia Liu, Jiejie Ge, Hongya Guan

**Affiliations:** 1 Department of Clinical Laboratory, Zhengzhou Orthopaedics Hospital, Zhengzhou, 450000 Henan China; 2 Department of Joint Surgery, Zhengzhou Orthopaedics Hospital, Zhengzhou, 450000 Henan China; 3 Department of Translational Medicine Center, Zhengzhou Central Hospital Affiliated to Zhengzhou University, Zhengzhou, 450007 Henan People’s Republic of China; 4 Department of Clinical Laboratory, Zhengzhou Central Hospital Affiliated to Zhengzhou University, Zhengzhou, 450007 Henan China

**Keywords:** Cancer, Risk factors

## Abstract

The present work aimed to establish a new model to accurately estimate overall survival (OS) as well as cancer-specific survival (CSS) of osteosarcoma. Osteosarcoma cases were collected from the Surveillance, Epidemiology, and End Results (SEER) database between 2004 and 2017 and randomized as training or validation sets. Then, the OS- and CSS-related variables were discovered through multivariate Cox regression analysis to develop new nomograms to predict the 1-, 3- and 5-year OS and CSS. Besides, consistency index (C-index), decision curve analysis (DCA), along with calibration curve were adopted for assessing the predicting ability of our constructed nomograms after calibrating for 1-, 3- and 5-year OS and CSS. Altogether, 1727 osteosarcoma cases were enrolled in the present study and randomly divided as training (n = 1149, 70%) or validation (n = 576, 30%) set. As shown by univariate as well as multivariate Cox regression analyses, age, grade, T stage, M stage, surgery, chemotherapy, and histological type were identified to be the adverse factors to independently predict OS and CSS among the osteosarcoma cases. Besides, based on results of multivariate Cox regression analysis, we constructed the OS and CSS prediction nomograms. The C-index in training set was 0.806 (95% CI 0.769–0.836) for OS nomogram and 0.807 (95% CI 0.769–0.836) for CSS nomogram. In the meantime, C-index value in validation set was 0.818 (95% CI 0.789–0.847) for OS nomogram, while 0.804 (95% CI 0.773–0.835) for CSS nomogram. Besides, those calibration curves regarding the 3- and 5-year CSS of our constructed nomogram were highly consistent between the predicted values and the measurements in the training set as well as the external validation set. Our constructed nomogram outperformed the TNM stage in prediction. Our constructed nomogram is facile, creditable, and feasible; it efficiently predicts OS and CSS for osteosarcoma cases and can assist clinicians in assessing the prognosis for individuals and making decisions.

## Introduction

Osteosarcoma is among the frequently occurring primary bone cancers, which particularly affects adolescents aged below 24 years and has an estimated annual prevalence of 0.34/100,000. Osteosarcoma is marked by its aggressiveness^1,2^, which manifests in the early lung metastasis and fast local invasion, resulting in high relapse and mortality rates^3^. After the emergence of adjuvant chemotherapy as well as limb salvage surgery, patients’ survival rate increased by 50% to about 70% in the most recent two decades^4,5^. Nonetheless, most osteosarcomas lead to disease- and treatment-associated morbidities or mortalities. Identification of the high-risk patients early is very important so as to offer a suitable clinical option or adjuvant therapy. Osteosarcoma can have a unique challenge in a clinical setting; as a result, it is urgently needed to establish the prognostic approaches to precisely estimate survival rates from osteosarcoma.

Currently, the TNM classification system is commonly employed to predict osteosarcoma prognosis^6^. Typically, the TNM classification system, released by the American Joint Commission on Cancer (AJCC), has been extensively employed to classify cancer patient survivals according to tumor invasion (T), regional lymph node (N) as well as distant metastasis (M)^7,8^. However, it is limited to evaluate cancer prognosis using the TNM classification system alone, which may not comprehensively assess the clinicopathological variables, including sex, age, race, or additional factors and is commonly employed to predict prognosis of osteosarcoma in extremities. It might not suitable to be used in axial location. Nomogram represents the statistical approach to determine the clinical event probabilities through taking into account those pre-weight values of all factors^9,10^. Recently, nomogram is extensively utilized for predicting diverse cancer survival^11–15^. In recent years, the massive data of cancer patients based on open-accessed data and bioinformatics methods make it possible for us to explore the independent risk factors for cancer prognosis^16–18^. The publically accessible SEER database includes cancer patient data across 18 registered sites that cover about almost 28% USA population^19,20^. This work was conducted to construct a creditable nomogram for predicting overall survival (OS) as well as cancer-specific survival (CSS) of osteosarcoma cases^21^; thus, assisting clinicians in providing better-customized treatment options to reduce the rate of metastasis and improve the survival rate of patients.

## Materials and methods

### Patients selection

The osteosarcoma cases in this work were selected from the SEER database, and their corresponding anonymous clinical data were extracted accordingly. The SEER*Stat software designed by the National Cancer Institute (version 8.3.6, https://seer.cancer.gov/seerstat/) was utilized, which covered the SEER 18 Regs custom data containing more therapeutic fields and the Nov 2018 Sub database (covering 2004–2016 data).

In this work, osteosarcoma cases conforming to the following inclusion criteria were selected: (1) Those with the diagnosis of osteosarcoma as the primary malignant tumor from 1983 to 2014 based on the International Classification of Diseases for Oncology [ICD-O] 9180–9187, 9192–9194^22^; (2) Those whose osteosarcomas were confirmed histologically; (3) Those with osteosarcoma in extremities (long/short bones in the four extremities) or at the axial location (skull, ribs, spine, and pelvis); (4) Those whose histological type was determined; (5) Those with estimated survival time or identified cause of mortality after they were diagnosed.

#### Exclusion criteria

Patients whose survival time was unavailable or unclear were excluded from this study.

The patient clinicopathological characteristics, such as age, gender, race, grade, histological type, tumor site, size, surgery, stage of surgery, chemotherapy, radiotherapy, and survival time, were harvested. As for age, the cases were divided as 0–24, 25–59 and > 59 years groups. The races were classified as black, white, or other (Alaskan Native/American Indian, Pacific/Asian Islander). The tumor sites were classified into an extremity (long/short bones in four extremities) or the axial location (skull, ribs, spine, and pelvis). Tumors were classified into three different sizes (≤ 89 mm, small; 89–139 mm, intermediate; and > 140 mm, large). Also, the low-grade tumors covered the moderately- and well-differentiated grades (namely, ICD-O-3 Grades I and II), whereas the high-grade tumors mainly included the poorly-differentiated and undifferentiated grades (namely, ICD-O-3 Grades III and IV). Meanwhile, surgery, chemotherapy, or radiotherapy were classified into yes or no.

### Statistical analyses

The specific processes of prediction model building and nomogram construction were as follow: Firstly, all patients were randomized as training or validation set in the ratio of 7:3. The development of the nomogram was performed using the training cohort, whereas the validation cohort was responsible for the validation. Secondly, the hazard ratios (HRs) and the 95% confidence intervals (CIs) were determined using univariate as well as multivariate Cox regression models and were used for assessing the contribution of every variable to OS or CSS independently. Thirdly, we introduced the significant variables identified from univariate analysis in the multivariate analysis to develop the nomograms for predicting 1-, 3- and 5-year OS and CSS. Additionally, another nomogram was also constructed on the basis of the TNM stage. Thereafter, the MedCalc software, version 15.2.0(MedCalc Software, Mariakerke, Belgium) was utilized for generating receiver operating characteristic (ROC) curves of these two nomograms, and the respective areas under the curve (AUC) values were determined. Moreover, C-index, as well as the calibration curve (1000 bootstraps resamples), were employed to evaluate the nomogram performance. Generally, C-index is between 0.5 and 1.0, with 0.5 indicating random chance, whereas 1.0 suggesting perfect discrimination. Also, net benefits of our constructed nomograms were evaluated by using decision curve analysis (DCA).

The R package (version 3.5.3, https://www.r-project.org/) and SPSS, version 24.0 (SPSS, Chicago, USA) were employed for statistical analysis. A difference of *P ≤ 0.05 (two-sided) indicated statistical significance.

## Results

### Demographic and pathologic characteristics

**Figure 1:**
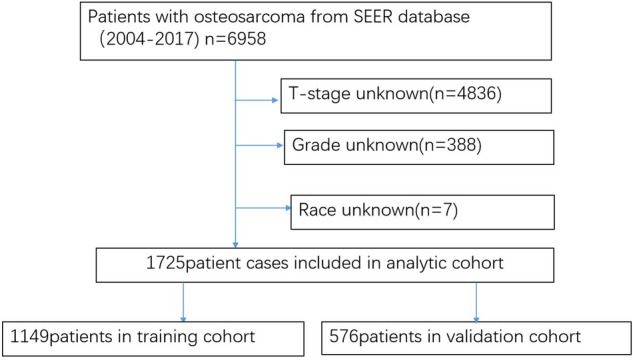
Schematic overview for patient identification.

Overall, 1725 cases were included into this work and randomized as training (n = 1149) or validation (n = 576) set. Figure 1 presents the patient inclusion process. Table 1 displays the demographic and pathological data of osteosarcoma cases. According to our results, patients aged 0–24 years (64.7%) showed the greatest morbidity of osteosarcoma, and most of the osteosarcoma cases were from white races (74.0%) and males (53.9%). As for the tumor site, osteosarcoma was most commonly located at the lower extremity (58.87%), followed by the primary axial location (28.81%). Besides, most patients were categorized into M0 stage (78.3%) and grade IV (51.4%). Besides, 86.6% of cases underwent surgical resection, while 79.8% underwent chemotherapy. Difference between the two data sets was not significant.

**Table 1:** Baseline characteristics of patients in the training and the validation cohort.

Variables	All patients (n = 1725) No. (%)	Training set (n = 1149) No. (%)	Validation set (n = 576) No. (%)	*P* value
Age(years)				0.951
0–24	978 (56.7)	651 (56.7)	327 (56.8)	
25–59	500 (29.0)	336 (29.2)	164 (28.5)	
≥ 60	247 (14.3)	162 (14.1)	85 (14.8)	
Sex				0.932
Male	929 (53.9)	622 (54.1)	312 (54.5)	
Female	796 (46.1)	527 (45.9)	264 (45.4)	
Race				0.758
Black	285 (16.5)	187 (16.3)	98 (17.0)	
White	1276 (74.0)	851 (74.1)	425 (73.8)	
Other	164 (9.5)	111 (9.7)	53 (9.2)	
Histologic type				
Osteogenic tumors	1616 (93.68)	1074 (93.5)	542 (94.10)	0.789
Fibrogenic tumors	109 (6.32)	75 (6.5)	34 (5.90)	
Tumor_site				0.825
Extremity	1253 (72.6)	837 (73.0)	416 (72.4)	
Axial	472 (27.4)	312 (27.0)	160 (27.6)	
Tumor_size				
0–89 mm	771 (44.7)	509 (44.5)	262 (45.5)	0.756
90–139 mm	483 (28.0)	322 (28.0)	161 (27.9)	
≥ 140	471 (27.3)	318 (27.5)	153 (26.6)	
Grade				0.925
Grade I–II	227 (13.2)	148 (12.5)	79 (13.4)	
Grade III–IV	1498 (86.8)	1001 (87.4)	497 (86.6)	
T stage				0.971
T1	633 (36.7)	425 (37.0)	208 (36.1)	
T2	869 (50.4)	577 (50.2)	292 (50.7)	
T3	42 (2.4)	29 (2.5)	13 (2.3)	
T4	181 (10.5)	118 (10.3)	63 (10.9)	
N stage				0.923
N0	1621 (94.0)	1080 (94.0)	541 (93.9)	
N1	37 (2.1)	25 (2.2)	12 (2.1)	
NX	67 (3.9)	44 (3.8)	23 (4.0)	
M stage				0.981
M0	1373 (81.3)	935 (81.5)	476 (82.6)	
M1	170 (18.7)	214 (18.4)	100 (17.4)	
Treatment				
Surgery				0.744
Yes	1494 (86.6)	998 (86.9)	496 (86.1)	
No	231 (13.4)	151 (13.1)	80 (13.9)	
Radiotherapy				0.977
Yes	180 (10.4)	118 (10.3)	62 (10.8)	
No	1545 (89.6)	1031 (89.7)	514 (89.2)	
Chemotherapy				0.802
Yes	1376 (79.8)	915 (79.6)	461 (80.0)	
No	347 (20.2)	234 (20.4)	115 (20.0)	

### Identification of OS and CSS-related factors based on training set

For identifying those prognosis-related factors, univariate as well as multivariate Cox regression analysis based on training set was conducted. Univariate Cox regression suggested that age at diagnosis, T stage, N stage, M stage, Grade, histological type, chemotherapy, surgery, and radiotherapy showed significant correlations with OS, whereas age, Grade, T stage, N stage, M stage, histological type, surgery, and radiotherapy showed tight correlations with CSS. Then, these above-screened variables were incorporated into multivariate analysis, which suggested that age, Grade, T stage, M stage histological type, and surgery independently predicted OS and CSS (Tables 2, 3).

**Table 2:** Multivariate analysis of cancer-specific survival (OS) rates in the training cohort.

	Univariate analysis		Multivariate analysis	
	HR (95% CI)	*P* value	HR (95% CI)	*P* value
Age				
25–59 vs. 0–24	1.711 (1.241–2.230)	< 0.001	1.794 (1.395–2.308)	< 0.001
≥ 60 vs. 0– 24	4.361 (3.056–6.223)	< 0.001	3.663 (2.706–4.958)	< 0.001
Sex				
Male vs. Female	1.055 (0.797–1.395)	0.167	1.076 (0.879–1.318)	0.477
Race				
White vs. Black	0.955 (0.663–1.375)	0.256	0.8721 (0.674–1.129)	0.256
Other vs. Black	0.679 (0.376–1.226)	0.230	0.500 (0.266–0.941)	0.192
Grade				
Grade III–IV vs. Grade II–I	4.416 (2.636–7.398)	< 0.001	5.267 (3.040–9.124)	< 0.001
Histologic type				
Osteogenic vs. Fibrogenic	0.788 (0.444–1.401)	0.418	0.934 (0.519–1.681)	0.024
Tumor_site				
Axial vs. extremity	2.336 (1.915–2.849)	< 0.001	1.976 (1.547–2.523)	< 0.001
Tumor_size				
90–139 mm vs. 0–89 mm	1.420 (1.108–1.819)	< 0.001	1.5153 (0.977–2.351)	0.064
≥ 140 vs. 0–89 mm	2.166 (1.718–2.731)	< 0.001	1.9471 (1.265–2.996)	0.002
T stage				
T2 vs. T1	1.220 (1.104–1.746)	0.004	1.607 (1.252–2.061)	< 0.001
T3 vs. T1	3.049 (1.896–4.905)	< 0.001	1.949 (1.124–3.380)	< 0.001
TX vs. T1	3.479 (2.539–4.575)	< 0.001	2.076 (1.479–2.914)	< 0.001
N stage				
N1 vs. N0	3.795 (2.340–5.908)	< 0.001	1.258 (0.759–2.084)	0.380
Nx vs. N0	1.723 (0.937–2.709)	0.006	0.658 (0.415–1.042)	0.118
M stage				
M1 vs. M0	4.042 (3.287–4.972)	< 0.001	3.730 (2.931–4.747)	< 0.001
Surgery				
Yes vs. no	0.177 (0.128–0.247)	< 0.001	0.399 (0.304–0.525)	< 0.001
Radiotherapy				
Yes vs. no	2.591 (2.001–3.340)	< 0.001	0.731 (0.465–1.147)	0.136
Chemotherapy				
Yes vs. no	0.749 (0.530–1.060)	0.052	0.700 (0.544–0.988)	0.013

**Table 3:** Multivariate analysis of f cancer-specific survival (CSS) rates in the training cohort.

	Univariate analysis		Multivariate analysis	
	HR (95% CI)	*P* value	HR (95% CI)	*P* value
Age				
25–59 vs. 0–24	1.549 (1.231–1.951)	< 0.001	1.780 (1.336–2.372)	< 0.001
≥ 60 vs. 0–24	4.361 (3.436–5.536)	< 0.001	3.259 (2.340–4.743)	< 0.001
Sex				
Male vs. Female	1.119 (0.920–1.360)	0.054	1.185 (0.93058–1.5103)	0.133
Race				
White vs. Black	0.856 (0.667–1.101)	0.405	0.8721 (0.674–1.129)	0.454
Other vs. Black	0.786 (0.376–1.174)	0.212	0.500 (0.266–0.941)	0.155
Grade				
Grade III–IV vs. Grade I–II	4.937 (2.627–9.277)	< 0.001	4.937 (2.627–9.277)	< 0.001
Histologic type				
Osteogenic vs. Fibrogenic	0.515 (0.230–1.156)	0.108	0.624 (0.273–1.423)	0.263
Tumor_site				
Axial vs. extremity	1.875 (1.463–2.403)	< 0.001	1.896 (1.409–2.552)	< 0.001
Tumor_size				
90–139 mm vs. 0–89 mm	1.809 (1.344–2.434)	< 0.001	1.473 (0.889–2.439)	0.132
≥ 140 vs. 0–89 mm	2.699 (2.037–3.574)	< 0.001	2.082 (1.276–3.397)	< 0.001
T stage				
T2 vs. T1	1.866 (1.404–2.488)	< 0.001	1.195 (1.252–2.061)	0.155
T3 vs. T1	4.279 (2.536–7.220)	< 0.001	1.894 (1.897–5.718)	0.062
TX vs. T1	3.608 (2.539–5.375)	< 0.001	1.076 (1.479–2.914)	0.813
N stage				
N1 vs. N0	4.318 (2.540–6.908)	< 0.001	1.474 (0.827–2.627)	0.172
Nx vs. N0	2.072 (1.282–3.407)	0.007	0.904 (0.511–1.599)	0.997
M stage				
M1 vs. M0	5.142 (4.092–6.462)	< 0.001	4.260 (3.250–5.582)	< 0.001
Surgery				
Yes vs. no	0.172 (0.138–0.214)	< 0.001	0.399 (0.304–0.525)	< 0.001
Radiotherapy				
Yes vs. no	2.783 (2.029–3.825)	< 0.001	0.731 (0.465–1.147)	0.445
Chemotherapy				
Yes vs. no	1.238 (0.884–1.734)	0.215	0.733 (0.544–0.988)	0.200

### Nomograms establishment and performance evaluation

**Figure 2:**
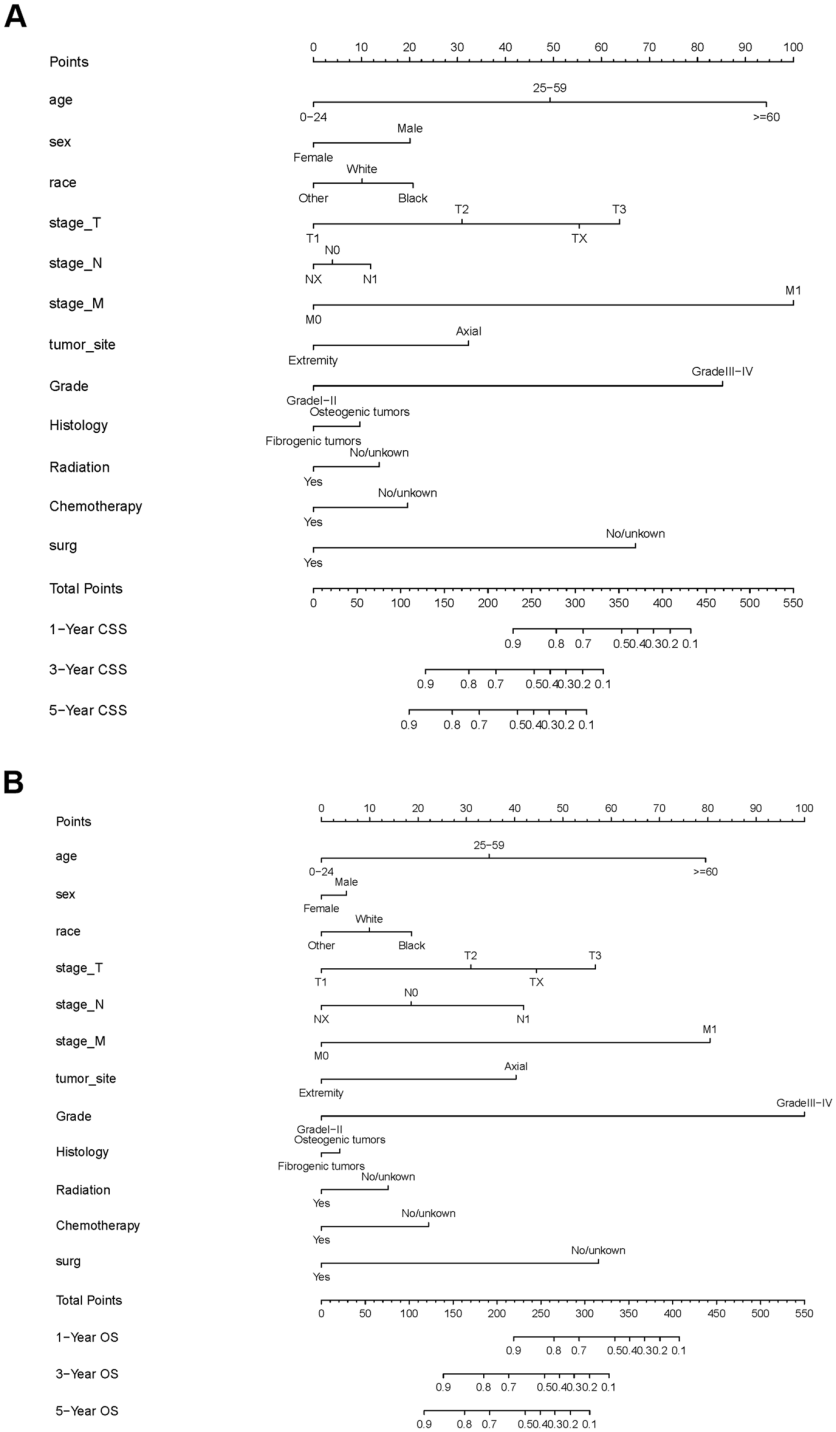
Nomogram predicting 1-, 3-, and 5-year overall survival (OS) and cancer-specific survival (CSS) rate of osteosarcoma patients. (**A**) OS rate; (**B**) CSS rate.

Different nomograms regarding OS and CSS were constructed based on multivariate Cox regression results (Fig. 2). Each variable was assigned a point according to HRs. The overall score of every variable was added, and its corresponding location in the total point scale was determined so as to obtain the probabilities of CSS at 1, 3 and 5 years. Disease grade contributed most to survival, as demonstrated in the CSS nomogram.

**Figure 3:**
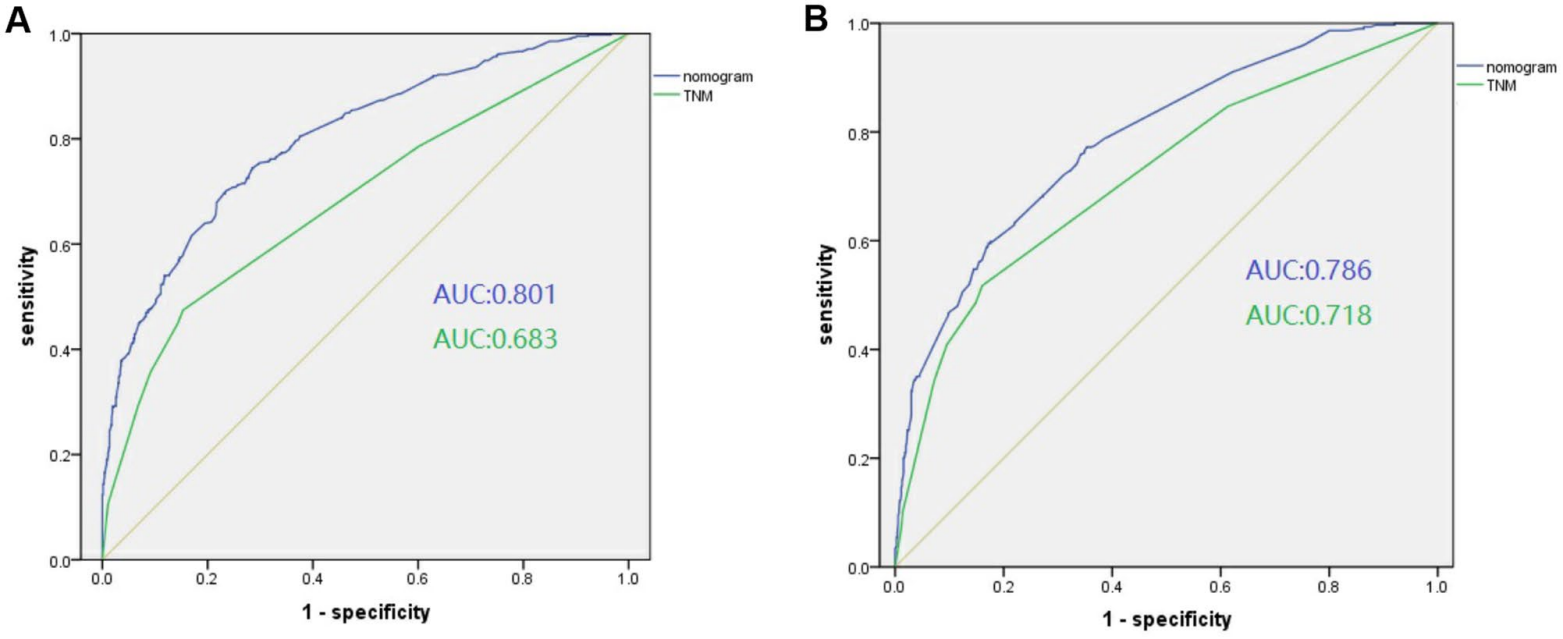
Receiver operating characteristic (ROC) and decision curve analysis (DCA) curves detect the predictive value of two nomograms in osteosarcoma prognosis. (**A**) ROC curve for overall survival (OS); (**B**) ROC for cancer-specifc survival (CSS).

**Figure 4:**
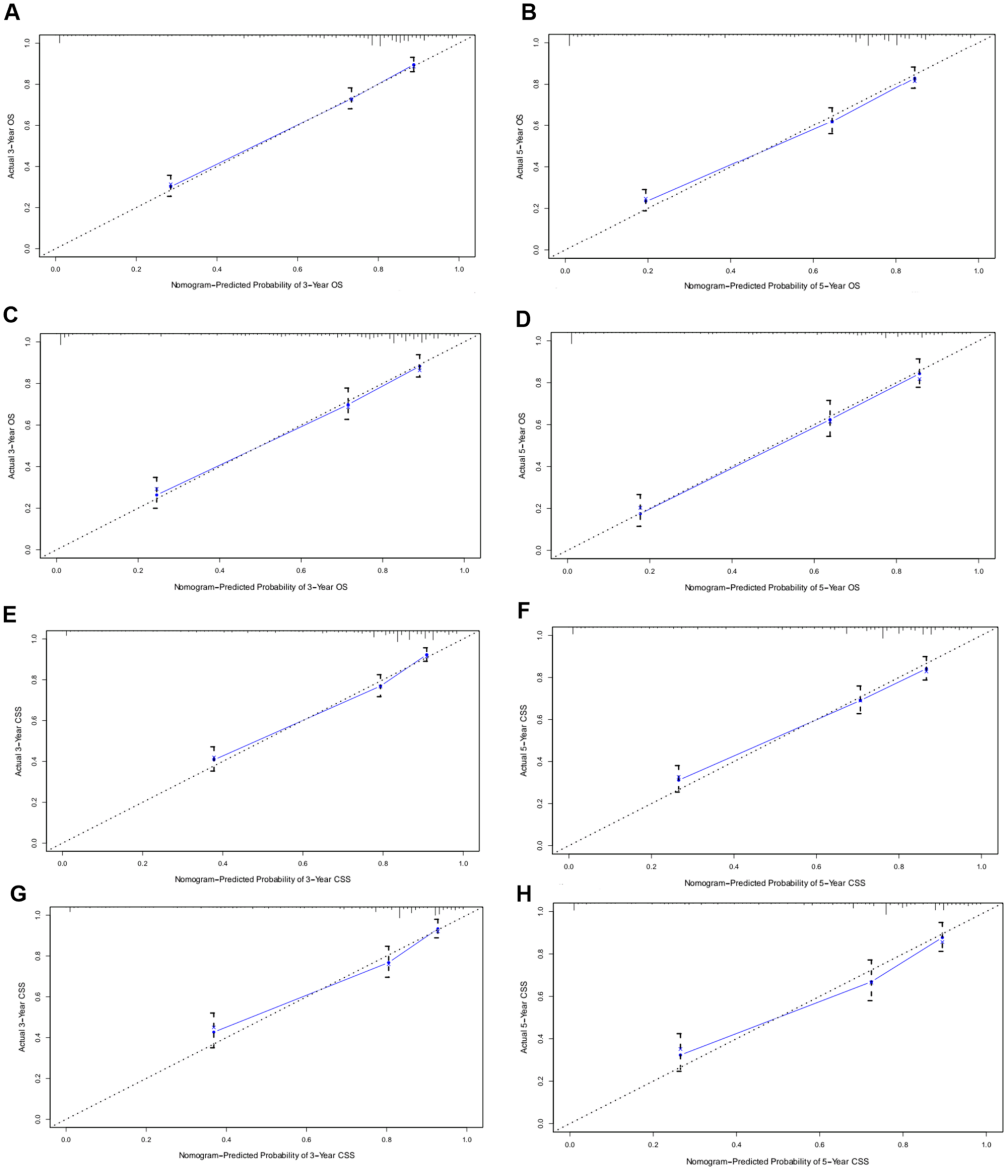
Calibration curves for 3- and 5-year survival. Calibration curves depict the calibration of each model in terms of the agreement between the predicted probabilities and observed outcomes of the training set (**A**–**D**) and validation set (**E**–**H**).

Thereafter, the time-dependent ROC curves regarding OS and CSS were analyzed. They displayed profoundly increased AUC values of our constructed nomograms (OS 0.801, 95% CI 0774–0.828; CSS 0.786, 95% CI 0.755–0.817) compared with those in the TNM stage (OS 0.683, 95% CI 0.650–0.717; CSS 0.718, 95% CI 0.682–0.754) of training set (Table 4 and Fig. 3A,B). For comparing the consistency between the estimated and real survival, we utilized C-index for verifying our constructed nomograms in the training set. Resultantly, C-indices of the constructed OS and CSS prediction nomograms (OS, C-index = 0.806; CSS, C-index = 0.807) increased in comparison to those in the TNM stage (OS, C-index = 0.686; CSS, C-index = 0.735). Table 4 shows a consistent trend detected in the validation set. This similarity in results in both study sets suggests the accuracy of the model based on our nomograms. Also, we calibrated the 3- and 5-year OS and CSS prediction nomograms in both sets (Fig. 4), which approached the optimal curve, displaying that values predicted by our nomograms were highly consistent with the real measurements in both sets.

**Table 4:** Comparison of area under the curve (AUC) between the nomogram and TNM stage in osteosarcoma patients.

Model	Training cohort			Validation cohort		
	C-index	95% CI	*P* value	C-index	95% CI	*P* value
CSS						
Nomogram	0.807	0.783–0.831	< 0.001	0.804	0.773–0.835	< 0.001
TNM stage	0.735	0.706–0.764	< 0.001	0.689	0.656–0.722	< 0.001
OS						
Nomogram	0.806	0.769–0.836	< 0.001	0.818	0.789–0.847	< 0.001
TNM stage	0.686	0.659–0.713	< 0.001	0.667	0.626–0.708	< 0.001

*AUC* area under the curve, *CI* confidence interval.

### Clinical applications

**Figure 5:**
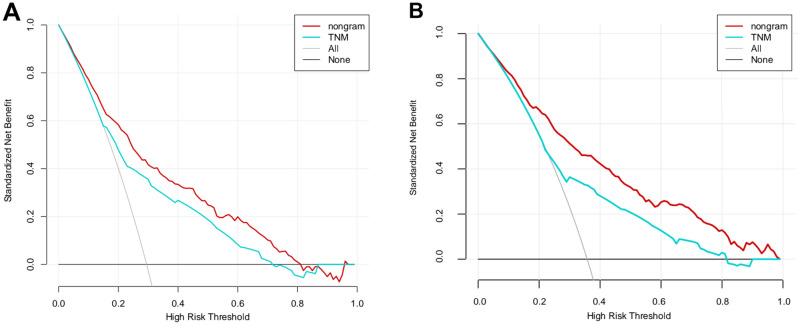
Decision curve analysis (DCA) curves detect the predictive value of two nomograms in osteosarcoma prognosis. (**A**) DCA for overall survival (OS); (**B**) DCA for cancer-specifc survival (CSS).

Besides, net benefits were also calculated by DCA for evaluating the clinical effectiveness of our constructed nomogram. Resultantly, the constructed nomograms showed increased clinical net benefits compared with those in the TNM stage in the wide range of OS (10–50%) (Fig. 5A,B).

## Discussion

Several prognosis-related factors may have a certain influence on the survival of osteosarcoma; however, the prognosis-related factors are not integrated previously. One individual prognosis index can lead to limited accuracy in predicting the prognosis for a patient. Nomogram has been the frequently used statistical approach to achieve high robustness and precision in predicting the probability of a patient’s overall survival. Kim et al.^23^. established a nomogram for predicting the metastasis risk among the nonmetastatic osteosarcoma cases that was validated to outperform the tumor necrosis rate or traditional AJCC classification system alone in predicting a distant metastasis (DM) of the tumor. Xia et al.^24^ established a nomogram for better estimating the prognosis for osteosarcoma cases that received surgery. Findings in the above studies could not be validated, so they had limited applicability in other populations because of the possible bias.

Kim and colleagues established a nomogram for predicting metastasis in patients with extremity osteosarcoma at Enneking stage IIB based on medical records from 91 cases that underwent surgery. Nonetheless, their study had a small sample size, so larger populations are required to validate the generalizability of their nomogram. In this study, the integrated and facile prognosis nomograms were constructed based on data collected from 1725 SEER-derived osteosarcoma patients so as to determine the OS and CSS at 3 and 5 years. Furthermore, our nomogram showed C-indices of 0.808 and 0.806, which were higher than most of the other nomograms for osteosarcoma.

Variables incorporated into the constructed nomogram were classified into 2 types of factors: (1) Clinical factors (such as age, race, grade, tumor size, tumor site, histological subtype, TNM stage), (2) Treatment-related factors (including surgery, chemotherapy, and/or radiotherapy). In the present work, most of our cases were under 24 years of age and occupied 56.7% and 56.8% in training and validation sets, respectively.

As depicted in Table 1, characteristics shown by many patients are: white, males, receiving surgery, had a tumor located at the extremities, and received chemotherapy. These findings corroborate those in previous studies. For the accurate selection of prognostic factors, univariate as well as multivariate Cox analyses were conducted for identifying factors to independently predict OS and CSS. According to our findings, age, tumor size, tumor site, TNM stage and grade, showed negative correlations with OS and CSS among the osteosarcoma cases, showing conformity with earlier findings^25–28^. M stage represents a factor that independently affects osteosarcoma patient prognosis. It is well known that distant metastasis of malignant tumors indicates a poor prognosis, which has been unanimously recognized by scholars^23^. However, due to the small number of baseline and outcome variables of N1 in the N staging, this variable could not become an independent prognostic factor influencing postoperative outcomes of patients with quadruped osteosarcoma when the multivariate COX regression model was fitted.

The TNM classification system has been commonly used to estimate the survival of osteosarcoma, but it just predicts the restricted osteosarcoma risk. In the present work, the practical nomograms were successfully established using 13 variables, including age, sex, race, grade, tumor size, tumor site, histological type, TNM stage, surgery, radiotherapy, and chemotherapy. As revealed by our results, our constructed nomograms outperformed the traditional TNM classification system in predicting patient survival (C-index: 0.806 vs. 0.686, 0.807 vs. 0.735), suggesting poor evaluative prognosis of the single TNM classification system.

Remarkably, our nomograms suggested that appropriate treatment must be given to extending patient survival. Amputation was adopted as the major treatment for high-grade osteosarcoma prior to the 1970s since adjuvant chemotherapy had not emerged at that time; this had severely affected the patient life quality and survival probability. Thanks to the emergence of adjuvant chemotherapy as well as limb salvage surgery, patient survival has risen by 50% to about 70%. In the current study, for an 18-year-old black patient who had high-grade osteosarcoma at T3N1M0 stage in the extremity (tumor size, 10.0 cm), receiving surgery and chemoradiotherapy could improve OS and CSS of the patient from 20 to 72%.

Compared to previous articles on osteosarcoma based on the SEER database, we have established a 1-year, 3-year, and 5-year overall survival (OS) and cause-specific survival (CSS) nomogram using the SEER database. By scoring various risk factors (age, race, gender, metastasis, pathological stage, surgery, chemotherapy), we comprehensively assess the survival outcomes of patients at 1 year, 3 years, and 5 years. We have also evaluated the clinical utility of our approach through net benefits analysis. However, this study suffered from some limitations. Firstly, a certain bias might have crept in due to its retrospective nature. Secondly, our nomograms were constructed on the basis of the large sample size, and the model was validated internally, but external validation was lacking. It was difficult to design an external validation study since osteosarcoma is not commonly seen. Yet, our constructed nomograms were able to effectively and precisely predict the survival for individual osteosarcoma cases.

## Conclusions

In this work, our constructed nomograms displayed great predicting ability. Findings in this study suggested that age, tumor site, tumor size, tumor grade, surgery, and TNM stage are factors that can independently estimate OS and CSS of osteosarcoma cases. Such factors are incorporated to establish nomograms to predict the survival of osteosarcoma cases. Our study presents a reliable and accurate method for predicting the survival of osteosarcoma patients. Additionally, the nomograms we developed can be effectively utilized to forecast the 1-, 3-, and 5-year overall survival (OS) and cancer-specific survival (CSS) rates for individual osteosarcoma cases. This valuable tool assists surgeons and clinicians in evaluating the likelihood of survival and determining the risk of mortality for each patient.

## Data Availability

The datasets used and/or analysed during the current study available from the corresponding author on reasonable request.
